# Effectiveness of rational emotive behavior therapy in reducing depression among undergraduate medical students

**DOI:** 10.1097/MD.0000000000032724

**Published:** 2023-01-27

**Authors:** Vera Victor-Aigbodion, Chiedu Eseadi, Zadrian Ardi, Abatihun Alehegn Sewagegn, Kennedy Ololo, Lazarus Bassey Abonor, Henry Egi Aloh, Temitope Ayodeji Falade, Offiong Asuquo Effanga

**Affiliations:** a Department of Educational Psychology, University of Johannesburg, Johannesburg, South Africa; b Department of Guidance and Counseling, Universitas Negeri Padang, Padang, Indonesia; c Institute of Education and Behavioral Science, Debre Markos University, Debre Markos, Ethiopia; d Department of Sociology, Alex Ekwueme Federal University, Ndufu, Alike Ikwo, Ebonyi State, Nigeria; e Department of Social Work, University of Calabar, Calabar, Cross River State, Nigeria; f Health Economics & Policy Research Unit, Department of Health Services, Alex Ekwueme Federal University, Ndufu, Alike Ikwo, Ebonyi State, Nigeria; g Department of Educational Foundations, University of Nigeria, Nsukka, Enugu State, Nigeria.

**Keywords:** depression, intervention, irrational beliefs, medical college, medical students, REBT

## Abstract

Studies reporting the incidence of depression among medical students have been on the increase. This research sought to determine whether rational emotive behavior therapy (REBT) can be applied to help manage depression in Nigerian undergraduate medical students.

**Methods:**

A randomized pretest/posttest control group design was used in this study. Ninety medical students with depression participated in the study and were assisted using the REBT depression manual. Using a mixed-model repeated measures analysis of variance, the researchers examined the intervention data.

**Results:**

The depressive symptoms and its associated irrational beliefs among medical students in the treatment arm were significantly altered by REBT intervention at posttest and this positive outcome was sustained at follow-up in contrast to the control arm.

**Conclusion:**

REBT intervention significantly improves medical students’ ability to overcome depression and irrational beliefs. Similar studies could be conducted in a variety of academic settings where these students can be found to expand the findings of this study.

## 1. Introduction

The aspiration to study medicine at the university is often the desire of many prospective students in developing regions. They create a mental picture of themselves in white coats as medical students and subsequently as physicians. However, behind the white coats are young undergraduate students battling psychological issues.^[1]^ In a study carried out at the Karolinska Institute Medical University, it was discovered that medical students had significantly higher prevalence of depressive symptoms than students in other fields, at 12.9%.^[2]^ The authors found that depression is linked to several stressors linked to a lack of feedback, concerns about future competence, an unwelcoming environment, and subpar teaching and learning techniques.^[2]^ Using DSM-III criteria, it was discovered that major depression occurs among medical students during the first 2 years of medical school.^[3,4]^ The Aga Khan University Anxiety and Depression Scale was used in a cross-sectional study at Nishtar Medical College, and the results showed a high prevalence of depression among medical students in their first, second, third, fourth, and final years: 45.86%, 52.58%, 47.14%, 28.75%, and 45.10%, respectively.^[5,6]^

Curriculum administration and design are also known to be among the main causes of depression in medical students.^[7,8]^ Additionally, a study reported that there are comorbidities in the causes of depression among medical students.^[1]^ It can include a polygamous family environment or an excessive academic workload that falls short of expectations.^[1]^ Medical students are also more likely to experience depression if they had recently gone through a traumatic event.^[9]^ In another study, the researchers discovered significant differences in depression rates among medical students, with female students experiencing depression at a higher rate.^[10]^ Similar studies conducted on medical students revealed that female medical students were more sensitive to the causes of depression than their male counterparts.^[2,11–13]^

In the last few decades, the cases of depression among Nigerian medical students have been on the increase with a prevalence rate of 23.3%.^[13]^ The authors discovered that female medical students preparing for their professional examination throughout the study period had greater levels of depression.^[13]^ Several factors contribute to varying degrees of depression among Nigerian medical students including frequent lecture rescheduling, high failure rates in Bachelor of Medicine and Bachelor of Surgery examinations, extensive distances from hostels to learning areas, and limited dormitory accommodation.^[14]^ More so, it was discovered that 4% of medical students were predisposed to moderate to severe depression in another Nigerian study, with gender and age not being substantially connected with depression.^[15]^

Years later, it was reported that male medical students were more prone to depression, with a 51.9% prevalence rate.^[16]^ The authors also discovered that fifth-year medical students had the largest proportion of depressed individuals (58.2%). However, according to a recent Nigerian study, the frequency of depression among Nigerian medical students was 15.1%.^[17]^ The study also found that depression was more prevalent in female medical students under the age of 22, those at a lower level of study, those with less social support, and those with a family history of depression.^[17]^ The primary goal of the present study is to investigate the effect of rational emotive behavior therapy (REBT) on depression among undergraduate medical students in Nigerian universities. The researchers expect that REBT would be useful in lowering depressive symptoms among the medical students. Also, due to the significant link between clients’ irrational beliefs and depressive symptoms, the researchers expects that the irrational beliefs associated with medical students’ depression will be significantly reduced by REBT intervention at posttest and be sustained at follow-up.

### 1.1. Theory of REBT in the context of depression

In REBT, irrational beliefs are considered to be substantial contributors to emotional disturbances like depression.^[18]^ The primary objective of REBT is to improve the occurrence of healthy and adaptable emotional and behavioral reactions by teaching patients to think more logically when faced with hardship.^[19]^ According to REBT, emotions and actions are not largely brought about by external events; rather, an individual’s beliefs about the events determine one’s emotional and behavioral responses.^[20]^ According to REBT, there are 4 main categories of illogical beliefs that influence depression. These include demandingness, self-downing, awfulizing, and low frustration tolerance.^[21–23]^ Demandingness is the irrational and unwavering insistence on things or people turning out the way the person wants them to. Awfulizing is the process of exaggerating a situation’s negative effects to an extreme degree to portray an already unfortunate event as terrible. Low frustration tolerance, also referred to as “I-can’t-stand-it,” is the admission of struggle accompanied by the assertion that the struggle is intolerable or nearly insurmountable. Self-downing, also known as depreciation, is the idea that someone generalizes based on their first attempt and assigns value to themselves in accordance with the outcome.^[23]^

The use of REBT techniques in treating psychological problems, such as depression and post-traumatic depression, has been shown to have significant advantages in the literature.^[24–27]^ REBT intervention programs can shield students from developing more severe mental health symptoms like major depressive disorders.^[28]^ In a recent study on the impact of REBT on depression management among students with learning disabilities, it was discovered that participants’ depression levels were significantly lower after receiving exposure to REBT during the posttest and follow-up than they were for participants who did not receive such exposure.^[29]^ The use of REBT for depression treatment cannot be overstated in view of studies showing a significant relationship between irrationality and depressive symptoms.^[30–35]^ Literature also suggests that a hybrid model of learning may be able to account for some of the cognitive and learning processes involved in the formation and maintenance of irrational beliefs and depression.^[32–35]^

## 2. Materials and method

### 2.1. Area of the study

This study took place in 3 public institutions in Nigeria’s South-South region, in the states of Edo, Delta, and Akwa Ibom in 2019. With a total population of 3,233,366 people, Edo State is one of Nigeria’s states with the highest levels of educational attainment. There are 4,112,445 people living in Delta state, an oil and agricultural producing state in Nigeria (men: 2,069,309; women: 2,043,136). The total land area of Delta state is 16,842 square kilometers (6503 sq mi). With a population of 5,482,200, Akwa Ibom State ranks as Nigeria’s highest oil-producing state in terms of crude oil and natural gas production. Currently, 7249 square kilometers of land make up Akwa Ibom State.

### 2.2. Design of the study

A randomized pretest/posttest control group design was used in this investigation.

### 2.3. Sample and sampling technique

**Figure 1.**
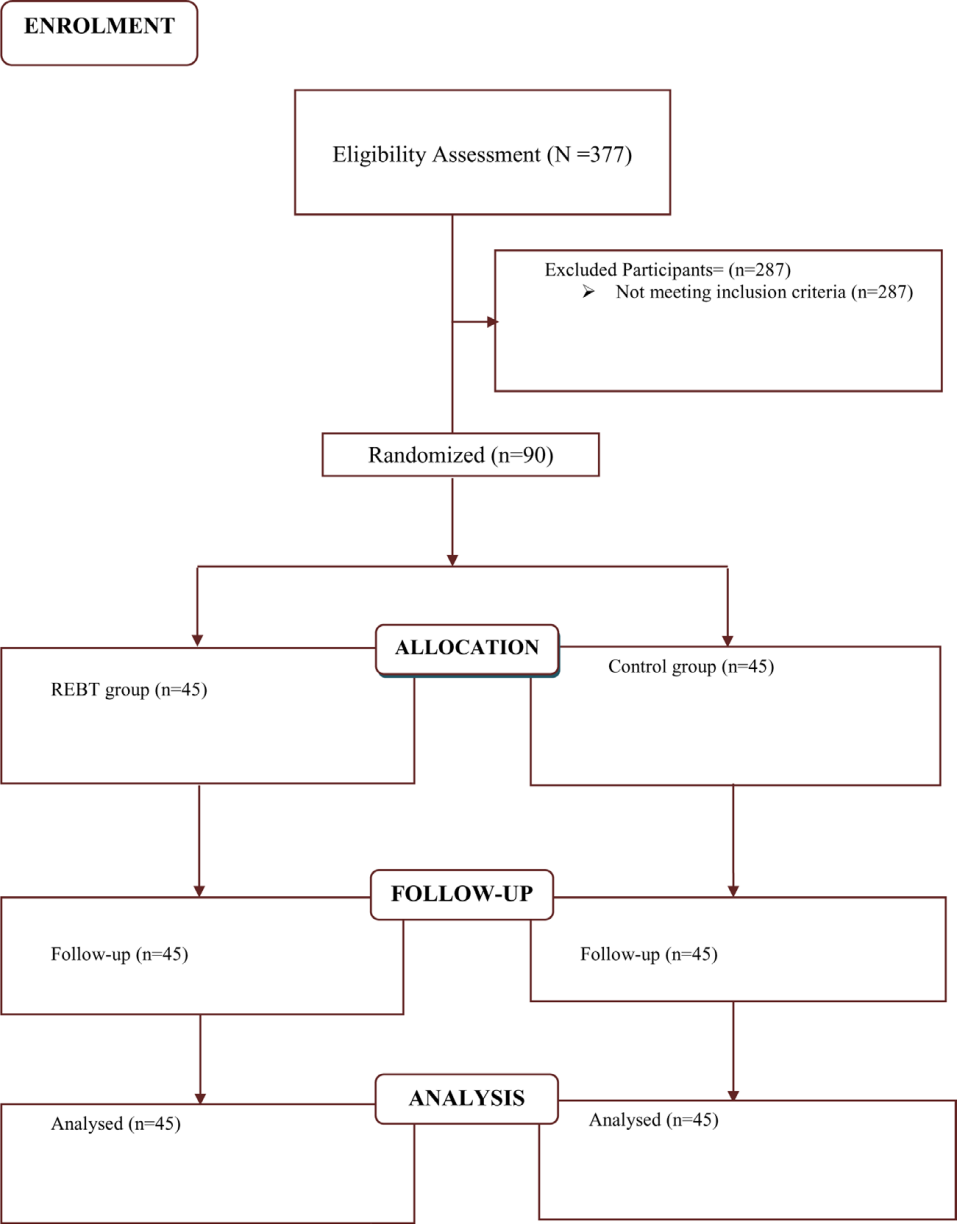
Participants’ flow diagram.

For this group intervention study, 90 medical students (N) are needed to maintain 80% power (medium effect size, d = .50; ICC=−.05).^[36]^ We divided N by the average number of members in each group (m = 15) for the 2 research arms, and used a 2-tailed test with an *α* level of .05. This yielded the total number of groups (G = 6) included in the study. With the use of Saghaei randomization program,^[37]^ group randomization produced 45 individuals for the intervention group and 45 people for the control group (see Fig. 1). The students’ bio-data dataset indicated that 53.3% were males while 46.7% were females. In addition, it was revealed that 66.6% were within the ages of 17 to 20, 27.7% were between the ages of 21 and 24, and 5.5% were between the ages of 25 and 28. Lastly, 60% of these students were in their first year while 40% were in their second year.

### 2.4. Method for selecting participants and characteristics

Ninety medical students with depression were the research participants. The participants were chosen from 3 Nigerian public universities following written informed consent. Following a random sample of first- and second-year medical Bachelor of Medicine and Bachelor of Surgery students from each of the chosen universities, the study was conducted.

### 2.5. Measures

The Zung Depression Inventory (ZDI) was used as a dependent measure in this study to determine participants’ depression levels.^[38]^ This 20-item self-reported questionnaire called the ZDI has already been used to reliably screen for depression in several settings. With a Cronbach *α* of 0.79, the ZDI is judged to have excellent psychometric qualities.^[3,39]^ Responses are gathered and given a score between 1 and 4 for each question, with a total score between 20 and 80. A score of <50 is thought to indicate the absence of depression, whereas a score of >50 indicated the presence of depression.^[38]^

The Beck Depression Inventory, version 2 (BDI-II) was used as a further dependent measure in this study to gauge participants’ depression levels.^[40]^ The goal of BDI-II was to uncover potential depression symptoms that ZDI was unable to detect. The BDI-II is a 21-item, self-administered questionnaire that measures depression severity on a 4-point scale from 0 to 3. The total score runs from 0 to 63, with scores between 0 and 13 being classified in the minimal range and 14 and 19 being considered mild, 20 to 28 is considered moderate, and 29 to 63 is considered severe.^[40]^

The Irrational Beliefs Inventory (IBI), created by Koopmans et al, was used as a supplementary dependent tool in this study.^[41]^ The IBI is a 50-item questionnaire that evaluates 5 types of irrational beliefs: the need for approval (7 things), worry (12 items), rigidity (14 items), emotional irresponsibility (7 items), and problem avoidance (10 items). Participants answered each question using a 5-point Likert scale (1 being strongly disagreed, and 5 being strongly agreed).

### 2.6. Description of intervention manual

The REBT depression manual served as the treatment plan for the intervention group. The REBT depression manual uses the rational-emotive and cognitive behavior therapy framework.^[42]^ This focuses on unreasonable notions such as demandingness, self-deprecation, awfulizing, and a low tolerance for frustration. In this study, medical students’ problematic beliefs were altered using REBT approaches. This intervention comprises of 20 sessions of a 14-week program that spans 2 weeks of follow-up meetings and 12 weeks of active therapy. Fifty minutes were allotted for each session.

### 2.7. Control condition

The researchers administered a placebo program to the members of the control group. Some therapists frequently employ the placebo program in their everyday work. A treatment guidebook for psychological wellbeing was modified by the researchers and used as the placebo manual. For the same number of weeks as the REBT intervention, the control program consisted of 20 sessions, each lasting 50 minutes. It worked as a placebo to occupy the members of the control group.

### 2.8. The therapists

Six psychologists, who were trained in cognitive behavioral approaches, including cognitive behavior therapy and REBT, provided the treatments. Each of them had a PhD in psychology, and they ranged in age from 35 to 50.

### 2.9. Integrity check

Six external assessors were tasked with monitoring the application of the REBT therapy and the control condition, respectively, to ensure that the therapists followed the manuals methodically. The assessors kept track of the participants’ attendance and oversaw each session throughout the treatment program.

### 2.10. Method of data analysis

SPSS version 22 and JASP statistical tools were used for the analyses. Using a mixed-model repeated measures analysis of variance, the researchers examined the intervention data. The pretest to posttest changes in the dependent variable is examined in the repeated measures model’s Time × Group interaction to see whether the intervention condition’s change is greater than the control condition’s change. Time is modeled as a categorical variable.^[44]^ Eta squared was used to calculate the intervention effect size (*η*^2^). The internal consistency of datasets for medical students’ depression was tested using Cronbach *α* reliability estimate.

The internal consistency coefficients of .644, .891, and .946 were established for ZDI, BDI–II, and IBI instruments. The reliability coefficients of these instruments were >0.6, indicating that the instruments were reliable. Furthermore, the temporary stability of the dataset was established using Pearson product moment correlation. The temporary stability coefficients of .739, .788, and .996 were computed for ZDI, BDI–II, and IBI instruments respectively. The high temporary stability coefficients show that the instruments are stable. This denotes a violation of equality of variance. The depression dataset as measured by ZDI was subjected to a sphericity test. The sphericity coefficient was not significant, [*x*^2^ (2) = 0.135, *P* = .225] denoting that the assumption of sphericity was not violated. Furthermore, there was a violation of the sphericity assumption in BDI–II and IBI datasets [*x*^2^ (2) = 10.377, *P* = .006] and [*x*^2^ (2) = 263.942, *P* < .001], hence, Greenhouse–Geisser was applied.

## 3. Results

Pretest mean ratings of depression among medical students are shown in Table 2 and were essentially the same for control and treatment arms. The mean rating of medical students significantly changed following the intervention (F [1, 87] = 257.533; *P* < .001, *η*^2^ = 0.253). Concerning time, there was a significant difference (F [2, 86] = 175.826; *P* ≤ .001, *η*^2^ = 0.328). And the interaction between intervention and time was significant (F[2, 86] = 91.790; *P* < .001, *η*^2^ = 0.171). Holm post hoc analysis was conducted. Concerning intervention, there was a difference in mean ratings of the treatment and control group (mean difference = −9.466, standard error = 0.590; *P* < .001). Based on time, significant differences existed between pretest and posttest (mean difference = 8.591, standard error = 0.704; *P* < .001), pretest and follow-up (mean difference = 12.978, standard error = 0.704; *P* < .001) and posttest and follow-up (mean difference = 4.387, standard error = 0.704; *P* < .001). Hence, the REBT intervention enhanced and sustained the reduction of depression among medical students as measured by the ZDI instrument.

**Table 1:** Summary of intervention process.

Phases	Wk	Sessions	Structure
Introduction phase	Wk 1–4	Sessions 1–8	• General assessment and conceptualization of the problem
			• Developing an empathic relationship with the clients.
			• Explaining REBT techniques and treatment
			• Stating the problem
			• The stated problem is tackled using the ABC (DEF) model of REBT
Middle phase	Wk 5–8	Sessions 9–16	• Supporting clients to understand the basis and similarities of their irrational thoughts and relationship with depression
			• Making efforts to replace clients’ irrational thoughts with rational beliefs
Final phase	Wk 9–12	Sessions 17–20	• Motivate clients to be enthusiastic to help themselves in the future
			• Explain the consequences of dependency and deterioration.
Follow-up phase	Wk 13–14	Sessions 21–22	• Follow-up counseling, motivation and assessment

REBT = rational emotive behavior therapy.

**Table 2:** Mean ratings and standard deviation of depression among medical students as measured by ZDI.

Time	Group	Mean	SD	N
Pretest	Control	61.36	3.56	44
	Treatment	62.16	4.94	45
Post-test	Control	62.21	5.51	44
	Treatment	44.13	5.35	45
Follow-up	Control	54.34	2.69	44
	Treatment	43.22	5.56	45

SD = standard deviation, ZDI = Zung Depression Inventory.

In Table 3, the mean ratings of depression as measured by the BDI-II instrument among medical students at pretest were significantly same for participants in control and treatment arms. After the intervention, there was a significant difference in the mean ratings of medical students in both groups (F [1, 87] = 217.143; *P* < .001, *η*^2^ = 0.252). Concerning time, there was a significant difference (F [2, 86] = 178.204; *P* < .001, *η*^2^ = 0.305). And the interaction between intervention and time was significant (F [2, 86] = 111.294; *P* < .001, *η*^2^ = .190). Holm post hoc test was conducted. Based on intervention, there was a difference in mean ratings of the treatment and control group (mean difference = −14.319, standard error = 0.972; *P* < .001). Based on time, significant differences existed between pretest and posttest (mean difference = 12.367, standard error = 1.021; *P* < .001), pretest and follow-up (mean difference = 18.978, standard error = 1.021; *P* < .001) and posttest and follow-up (mean difference = 6.611, standard error = 1.021; *P* < .001). Hence, the REBT intervention enhanced and sustained the reduction of depression among medical students as measured by the BDI-II instrument.

**Table 3:** Mean ratings and standard deviation of depression among medical students as measured by BDI–II.

Time	Group	Mean	SD	N
Pretest	Control	40.69	3.77	45
	Treatment	42.42	9.85	45
Post-test	Control	43.47	7.31	45
	Treatment	14.91	7.02	45
Follow-up	Control	30.64	5.51	45
	Treatment	14.51	8.41	45

BD–II = Beck Depression Inventory, version 2, SD = standard deviation.

Table 4 shows that the mean scores of medical students exposed to treatment and control conditions did not significantly differ at pretest according to the IBI. After the intervention, there was a significant difference in the mean ratings of medical students in both groups (F [1, 87] = 179.748; *P* < .001, *η*^2^ = 0.334). Concerning time, there was a significant difference (F [2, 86] = 112.576; *P* < .001, *η*^2^ = 0.145). And the interaction between intervention and time was significant (F [2, 86] = 190.197; *P* < .001, *η*^2^ = 0.244). Holm post hoc test was conducted. Based on intervention, there was a difference in mean ratings of the treatment and control group (mean difference = −44.193, standard error = 3.296; *P* < .001). Based on time, significant differences existed between pretest and posttest (mean difference = 30.711, standard error = 2.373; *P* < .001), pretest and follow-up (mean difference = 30.956, standard error = 2.373; *P* < .001) but not between posttest and follow-up (mean difference = 0.244, standard error = 2.373; *P* = .918). Hence, the REBT intervention enhanced and sustained the reduction of irrational beliefs among medical students as measured by the IBI instrument.

**Table 4:** Mean ratings and standard deviation for irrational beliefs among medical students as measured by IBI instruments.

Time	Group	Mean	SD	N
Pretest	Control	96.89	12.41	45
	Treatment	106.13	24.04	45
Post-test	Control	106.13	24.04	45
	Treatment	35.47	16.59	45
Follow-up	Control	106.13	24.04	45
	Treatment	34.98	17.78	45

IBI = Irrational Beliefs Inventory, SD = standard deviation.

## 4. Discussion

The study found that REBT significantly enabled medical students to manage depression and overcome associated irrational beliefs. This finding is in line with a previous study that participants’ depressive symptoms significantly reduced during posttest and follow-up using REBT intervention in a sample of 48 Nigerian students.^[30]^ Also, in a clinical control trial conducted among a sample of 88 depressed Romanian youths, it was found that significant decreases in self-reported depression occurred during the post-treatment under REBT conditions.^[45]^ Similar to this, using a group randomized controlled trial design, it was discovered that the REBT group significantly reduced their depression scores both at the post-treatment and follow-up evaluations when compared to the no-intervention control group in a sample of 65 depressed undergraduate students.^[26]^ Following a REBT therapy intervention, a significant decrease in participants’ illogical beliefs was observed by another study,^[46]^ which is consistent with the findings of the current study. Another study reported that participants’ irrational beliefs decreased significantly following a treatment intervention that used REBT.^[47]^ It is also interesting to mention that there have been a number of previous studies that have demonstrated the clinical importance of REBT in reducing irrational beliefs and fostering rational beliefs among participants, and these studies are consistent with the current study.^[48–50]^

There are some other studies whose findings support our current results. For instance, in a quasi-experimental study using the REBT group for depression treatment among 60 depressed Malaysian teenagers, the authors found out that REBT structured counseling group was effective in bringing down the psychological variables of depression with no gender difference.^[51]^ Additionally, a quasi-experimental pretest-posttest study reported that REBT was effective in lowering depression in patients with hypertension during the posttest and follow-up period.^[52]^ In another previous study among 23 education undergraduates with the major depressive disorder at a Nigerian university, the authors found that REBT was successful in lowering depression ratings among the students.^[53]^ More so, some researchers conducted a study to determine the cost-effectiveness and cost-utility of REBT in treating depression among a Romanian sample of 170 depressed clients.^[54]^ The researchers discovered that the depressed patients’ average depression scores fell from 31.1 prior to therapy to 9.7 after REBT was finished.^[54]^ Another quasi-experimental study that included 45 divorced women in Iran who were randomly assigned to experimental and control groups by convenience sampling revealed a significant difference between the mean depression scores of the experimental and control groups, indicating that REBT may help divorced women with their depression and irrational beliefs.^[55,56]^ A pretest-posttest approach was employed to investigate the efficacy of REBT on depressed female Iranian adolescents and the results were positive^[57]^ and in line with the present research findings.

### 4.1. Study limitations and implications

One of study’s limitations is that it only included undergraduate medical students from 3 public institutions in Nigeria’s south-south region; hence, medical students from private universities and other public universities were not considered. As such, the results should be applied with caution given its limited scope. Another limitation is the exclusive use of quantitative tools to address depression and irrationality in medical students which we think are unable to offer deep insights into the lived experiences of these students. Therefore, the use of qualitative tools to examine depression and irrational beliefs in medical students is recommended in future studies in order to provide greater understanding of these psychological concerns from the perspective of these students and further influence psychological treatment options that could be employed in future research.

One implication of this study is that in order to improve medical students’ mental health, university counseling centers must work towards providing equitable mental health services to undergraduate students. They also need to implement therapeutic innovations in their mental health counseling services which would include REBT interventions. To lower the prevalence of irrational views among medical students, therapists must continue to emphasize the development of logical beliefs amongst these students. This is because eliminating irrational beliefs is crucial for lowering depressed symptoms in medical students and improving their psychological well-being.

## 5. Conclusion

We applied the REBT intervention among university undergraduate medical students to examine whether this intervention helps to alleviate depressive symptoms and related irrational beliefs in these students. The study showed that REBT intervention is significantly effective in enabling medical students to manage depression at post-treatment and this outcome was sustained at follow-up. The irrational beliefs associated with medical students’ depression were significantly altered by REBT at posttest and follow-up. Therefore, similar studies should be conducted in a variety of academic settings in which these students are found, to expand the findings of this study.

## Author contributions

**Conceptualization:** Vera Victor-Aigbodion, Chiedu Eseadi, Zadrian Ardi, Abatihun Alehegn Sewagegn, Kennedy Ololo, Lazarus Bassey Abonor, Henry Egi Aloh, Temitope Ayodeji Falade, Offiong Asuquo Effanga.

**Data curation:** Vera Victor-Aigbodion, Chiedu Eseadi, Zadrian Ardi, Abatihun Alehegn Sewagegn, Kennedy Ololo, Lazarus Bassey Abonor, Henry Egi Aloh, Temitope Ayodeji Falade, Offiong Asuquo Effanga.

**Formal analysis:** Vera Victor-Aigbodion, Chiedu Eseadi, Zadrian Ardi, Abatihun Alehegn Sewagegn, Kennedy Ololo, Lazarus Bassey Abonor, Henry Egi Aloh, Temitope Ayodeji Falade, Offiong Asuquo Effanga.

**Funding acquisition:** Vera Victor-Aigbodion, Chiedu Eseadi, Zadrian Ardi, Abatihun Alehegn Sewagegn, Kennedy Ololo, Lazarus Bassey Abonor, Henry Egi Aloh, Temitope Ayodeji Falade, Offiong Asuquo Effanga.

**Investigation:** Vera Victor-Aigbodion, Chiedu Eseadi, Zadrian Ardi, Abatihun Alehegn Sewagegn, Kennedy Ololo, Lazarus Bassey Abonor, Henry Egi Aloh, Temitope Ayodeji Falade, Offiong Asuquo Effanga.

**Methodology:** Vera Victor-Aigbodion, Chiedu Eseadi, Zadrian Ardi, Abatihun Alehegn Sewagegn, Kennedy Ololo, Lazarus Bassey Abonor, Henry Egi Aloh, Temitope Ayodeji Falade, Offiong Asuquo Effanga.

**Project administration:** Vera Victor-Aigbodion, Chiedu Eseadi, Abatihun Alehegn Sewagegn, Lazarus Bassey Abonor, Henry Egi Aloh, Offiong Asuquo Effanga.

**Resources:** Zadrian Ardi, Kennedy Ololo.

**Software:** Chiedu Eseadi, Zadrian Ardi, Abatihun Alehegn Sewagegn, Kennedy Ololo, Lazarus Bassey Abonor, Henry Egi Aloh, Temitope Ayodeji Falade, Offiong Asuquo Effanga.

**Supervision:** Vera Victor-Aigbodion, Chiedu Eseadi, Zadrian Ardi, Kennedy Ololo, Temitope Ayodeji Falade.

**Validation:** Vera Victor-Aigbodion, Chiedu Eseadi, Zadrian Ardi, Abatihun Alehegn Sewagegn, Kennedy Ololo, Lazarus Bassey Abonor, Henry Egi Aloh, Offiong Asuquo Effanga.

**Visualization:** Zadrian Ardi, Lazarus Bassey Abonor, Henry Egi Aloh.

**Writing – original draft:** Vera Victor-Aigbodion, Chiedu Eseadi, Zadrian Ardi, Abatihun Alehegn Sewagegn, Kennedy Ololo, Lazarus Bassey Abonor, Henry Egi Aloh, Temitope Ayodeji Falade, Offiong Asuquo Effanga.

**Writing – review & editing:** Vera Victor-Aigbodion, Chiedu Eseadi, Zadrian Ardi, Abatihun Alehegn Sewagegn, Kennedy Ololo, Lazarus Bassey Abonor, Henry Egi Aloh, Temitope Ayodeji Falade, Offiong Asuquo Effanga.
